# Infiltration tendency of internal mammary lymph nodes involvement in patients with breast cancer: anatomical characteristics and implications for target delineation

**DOI:** 10.1186/s13014-019-1412-z

**Published:** 2019-11-21

**Authors:** Yujie Wang, Weixiang Qi, Haoping Xu, Miao Zhang, Yimin Han, Jiayi Chen, Cheng Xu

**Affiliations:** 1 Department of Radiation Oncology, Ruijin Hospital, Shanghai Jiaotong University, School of Medicine, 197# Ruijin Er Road, Outpatient Building, Shanghai, China; 2 Department of Nuclear Medicine, Ruijin Hospital, Shanghai Jiaotong University, School of Medicine, 197# Ruijin Er Road, Building 7, 3rd floor, Shanghai, China

**Keywords:** IMN, IMV, Anatomical features, Infiltration tendency, CTV delineation

## Abstract

**Background:**

Despite increasing clinical data suggest that internal mammary node (IMN) irradiation would improve local-regional control and overall survival in breast cancer, its role remains controversial due to increased risk of cardiac and pulmonary toxicity. The current study aims to determine the high risk areas of IMN metastases by collecting and analyzing the axial imaging of IMN involvement, in order to optimize IMN delineation for breast cancer.

**Methods:**

Breast cancer patients with IMN involvement were retrospectively identified from single-center database. All available imaging modalities including thoracic CT, breast MRI, ultrasound and PET/CT were used to diagnose IMN metastases. Anatomical characteristics from axial imaging, including distribution of involved ribs and distance from the internal mammary vessels (IMV), were collected for each metastatic IMN. What’s more,the natural infiltration tendency of IMNs from IMV was calculated in this study.

**Results:**

In total, 83 metastatic IMNs from 70 breast cancer patients (initial diagnosed:34 and recurrence: 36) were located from axial CT image in this study. The second intercostal space was the most likely involved in patients with single(*n* = 35, 53.0%) and multiple intercostal space (*n* = 31, 47.0%) involvement. The percentage of including IMN with a 5 mm, 6 mm and 7 mm medial/lateral distance to the IMV were 75.9% (63/83), 89.2.6% (74/83) and 92.3% (77/83) respectively. While in maximal dorsal/ventral distance, nearly 95% of the nodes were encompassed into 6 mm depth to the IMV. Over 65% of IMN adenopathy (32/49,65.3%) were found to have a growth direction close to the sternum. By retrospective reviewing diagnostic reports, MRI demonstrated a high diagnostic performance in diagnosis of IMN disease (90.3%, 28/31), while CT had a higher misdiagnosis rate (22/63, 34.9%). The diagnostic efficiency of IMN could be improved if different methods were combined.

**Conclusions:**

For patients with indications of prophylactic IMN irradiation, a 7 mm medial and 6 mm dorsal distance to the IMV on axial CT would be optimal to cover the clinical volume of IMN; and it would be reasonable to extend clinical tumor volume (CTV) coverage towards sternum for patients with evident IMN disease. Multi-imaging modalities are recommended to improve the diagnostic specificity and sensitivity of IMN metastases.

## Backgroud

As a primary lymphatic draining site in breast, increasing attention has been paid to the internal mammary lymph node chain along with the axillary lymph nodal basin. Evidence from radical (Halsted) mastectomy in the 1950s has validated that nearly one third of breast cancer patients would experience IMN involvement on surgical biopsies [1, 2]. Modern Lymphatic mapping techniques such as isosulfan blue dye and technetium-99 labeled sulfur colloid or the combination of both, has confirmed that approximately 25% (ranges: 13 to 37%) of breast cancer patients have primary IMN drainage. In addition, the position of the tumor in the media and the status of positive axillary lymphnode are independent risk factors for IMN involvement [3–6]. It was also established that patients with IMN metastases have a worse prognosis than patients who do not, independent of their axillary status [7–11]. Therefore, how to appropriately manage IMN chain becames a hot issue in breast cancer treatment.

Radiotherapy, after the surgical intervention, has been a widely accepted standard of care in the management of breast cancer for appropriate indications, as shown by numerous randomized trials and meta-analyses which confirm that RT could significantly decrease the risk of LRR and improve overall survival. EBCTCG meta-analyses, along with EORTC 22922–10,925, MA.20, and DBCG-IMN clinical trials have shown a significanlt benefit of local-regional control and survival in positive axillary lymph nodes (ALNs) or high-risk ALN negative breast cancer patients who receiving regional node irradiation (RNI) [12–16]. Based on these practice-changing randomized controlled studies, internal mammary lymph node irradiation was delivered as part of post-mastectomy or post-lumpectomy radiation therapy in addition to systemic therapy in some subgroup of breast cancer with high-risk features.

Despite these positive evidence, to include IMN as part of RNI or not has not reached a consensus. One reason could be its anatomical position immediately adjacent to heart and lungs. Thus IMN irradiation would increases the dose and volume of normal tissue radiated, especially in left-sided patients might offset the therapeutic effect of IMN irradiation [17–19].Additionally,anthracycline-based chemotherapy and concurrent administration of trastuzumab with adjuvant radiation therapy for HER2-positive patients may also exacerbate heart toxicity [19–22] .

Given the anatomical location of the IMNs and adjuvant syetem regimens, inclusion of IMN in the RNI would inevitably increase the complexity of the treatment planning [17]. And current recommendations for CTV of IMN remain controversial. For example, the PROCAB, DBCG, and ESTRO groups suggest a uniform 5-mm expansion on the IM vessels, while RTOG guideline encompasses the IMV only [23–26]. As a result, it would be helpful to optimally cover the clinical volume of IMN and minize normal tissue volume exposure in the era of intensity-modulate radiotherapy (IMRT).

What is worse, within the image characteristics of regional nodes involvement in breast cancer, data on IMN involvement is relatively scarce than others. Despite the lower frequency of IMN metastasis compared to ALN, lack of sensitivity in radiological early detection and no universal consensus for abnormal IMN on imageological diagnosis also explain to the insufficient report [27–30].

The aim of the current study is to locate and map the involved IMN in order to improve the existing recommendation of IMN contouring guideline. Also, by search on radiological system using possible confounding description, to reveal the existence of under-diagnosis of IMN metastasis in real world.

## Methods

Patients with diagnostic breast cancer and complete imaging records for any possible internal mammary nodes involvement were retrospectively identified from breast cancer database at a single institution. Strings or terms for searching, including “internal mammary node”, “IM node”, parasternal mass/lump or chest-wall mass/lump, were used to identify imaging slices with abnormal IMN(s) as documented by a breast radiologist. Image methods included in the search contain ultrasound, thoracic CT, breast MRI, and PET/CT. Each imaging slice with possible abnormal IMN(s) was reviewed by a senior radiologist and a radiation oncologist independently on the basis of suspicious radiographic appearance including shape, size, contrast enhancement, MRI signal and fluorodeoxy-glucose avidity, and a final determination was reached on the consensus. The axial CT image with the most evident mass in the internal mammary node region would be selected for subsequent calculation and measurement if multiple records was retrieved with the same patient. IMN greater than 5 mm in short axis was defined as metastasis in this study.

**Fig. 1:**
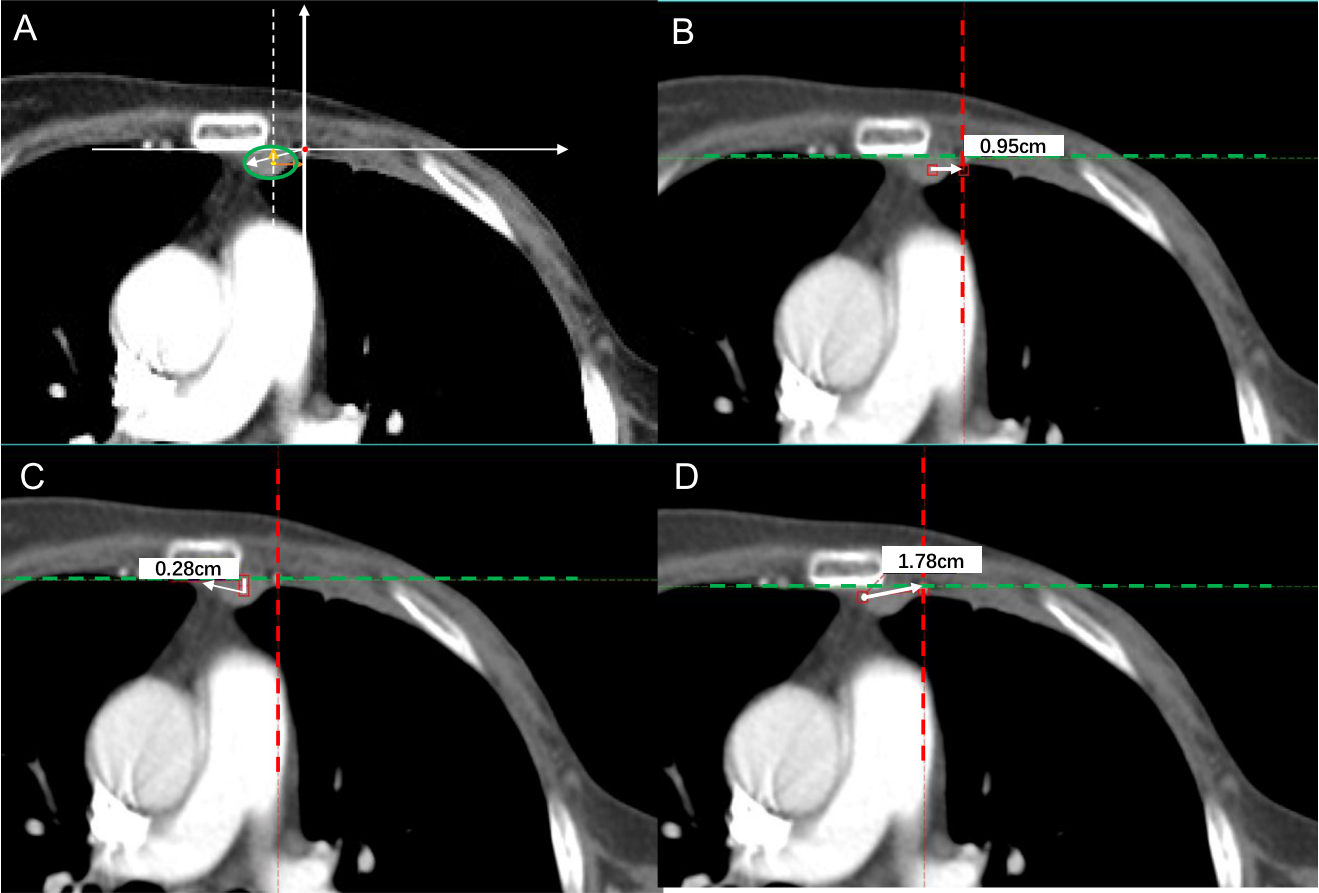
Schematic diagram of the IMN extending measurement. **a** Green circle: internal mammary lymphadenopathy. Red dot: internal mammary vessel; Arrows: distance between internal mammary vessel and internal mammary lymph node in medial direction, dorsal direction and longest distance. **b** Example of measurement in medial/lateral axis; **c** Example of measurement in dorsal axis; **d** Example of longest distance between tumor margin and internal mammary vessels

For each patient, anatomical characteristics from axial CT image including the number and size of the involved IM lymph node(s), intercostal space location(s) were documented, and distance from the center of node to IMV were measured for each metastatic node. To analyze the nature extending direction of IMNs, we anchored the ipsilateral IMV as center and measured the distance from the farthest point of the lesion to the vessel. On the axial CT image, the position of the IM vessel was marked firstly, and then the maximum distance of the mass from different directions in each quadrant was measured and spotted to calculate the probability of falling within each quadrant. Figure 1 displays the schematic diagram of the measurement.

Chi-square test was employed to compare the differences between these spots located in the four quadrants. *P* < 0.05 was considered to be of statistical significance. Statistical analysis was performed with SPSS software.

## Results

### Patient characteristics

In total, 83 metastatic IMNs in 70 breast cancer patients (four patients with bilateral breast cancer) were identified with the aforementioned diagnostic criteria. The IMNs were located from axial CT image in this study and seven cases were measured from PET/CT series. Thirty-four patients were identified IMN metastases at initial diagnosis and 36 recurrent with or without distance metastasis, among whom four patients were lack of initial diagnostic imaging information. Seventeen patients also had axillary regional nodes involvement concurrently, 14 patients had primary distant metastasis at initial diagnosis. Among the patients with relapse or metastases, only nine patients had IMNs recurrence alone, while the rest patients had concurrent local-regional recurrence and/or metastases. Four patients had pathologically confirmed IMN disease via extend radical mastectomy. Biopsy of the IMN was not routine as most patients had simultaneously confirmed metastases in the axillary or the supraclavicular lymph nodes that were sampled by fine-needle aspiration (FNA). The management of IMNs recurrence was performed at the physician’s discretion, 10 patients did undergo FNA of the suspected IMN under the guidance of ultrasound or CT, and cytology confirmed metastatic carcinoma were diagnosed in all of these patients. The general characteristics of the study group were shown in Table 1.

**Table 1:** General characteristics of study group

Characteristics	Initial diagnosis with IMN involved(*n* = 34)	Recurrence with IMN involved (*n* = 36)	All patients (*n* = 70)*
ER status, n(%)			
Positive	20 (58.8)	25 (69.4)	45 (64.3)
Negative	12 (35.3)	10 (27.8)	22 (31.4)
Unknown	2 (5.9)	1 (2.8)	3 (4.3)
PR status, n(%)			
Positive	13 (38.2)	19 (52.8)	32 (45.7)
Negative	18 (52.9)	15 (41.7)	33 (47.1)
Unknown	3 (8.8)	2 (5.6)	5 (7.1)
HER2 status, n(%)			
Positive	12 (35.3)	11 (30.6)	23 (32.9)
Negative	20 (58.8)	22 (61.1)	42 (60.0)
Unknown	2 (5.9)	3 (8.3)	5 (7.1)
T stage, n(%)			
Tis	1 (2.9)	0 (0)	1 (1.4)
T1	4 (11.8)	10 (27.8)	14 (20.0)
T2	13 (38.2)	17 (47.2)	30 (42.9)
T3	4 (11.8)	2 (5.6)	6 (8.6)
T4	8 (23.5)	1 (2.8)	9 (12.9)
Tx	4 (11.8)	6 (16.7)	10 (14.3)
N stage, n(%)			
N0	0 (0)	19 (52.8)	19 (27.1)
N1	0 (0)	6 (16.7)	6 (8.6)
N2	1 (2.9)	3 (8.3)	4 (5.7)
N3	33 (97.1)	6 (16.7)	39 (55.7)
Nx	0 (0)	2 (5.6)	2 (2.9)
M status, n(%)			
M0	20 (58.8)	15 (41.7)	35 (50.0)
M1	14 (41.2)	21 (58.3)	35 (50.0)

*Including four patients with bilateral breast cancer

Four patients had missing information

### Anatomic characteristics of IMN metastases

#### IMNs in relation to intercoastal spaces

The number of metastatic nodes located in the 1st, 2nd, 3rd and 4th intercostal space was 28 (42.4%), 58 (87.9%), 20 (30.3%) and 4(6.1%) respectively. The second intercostal space was the most likely invaded among patients not only with single(*n* = 35, 53.0%) but also with multiple intercostal spaces (*n* = 31, 47.0%) involvement. Only four IMNs were distributed in the 4th intercostal space and one was continued from the subclavian vein. Table 2 shows the intercostal locations of the study group.

**Table 2:** Involved ribs location and local-regional relapse of study group

	Initial diagnosis n = 34		Recurrent *n* = 32*		Summary *n* = 66(%)
Involved region	M0	M1	M0	M1	–
IMN (pts/No)	20	14	14	18	66
1st intercostal	5	10	6	7	28 (42.4)
2nd intercostal	17	13	11	17	58 (87.9)
3rd intercostal	3	4	4	9	20 (30.3)
4th intercostal	0	2	0	2	4 (6.1)
Single intercostal	15	4	7	9	35 (53.0)
≥1 intercostal	5	10	7	9	31 (47.0)
Local recurrence	–	–	9	8	17 (53.1)**
ALN I	19	13	0	3	35 (53.0)
ALN II	13	10	1	7	31 (47.0)
ALN III	9	8	1	6	24 (36.4)
SCN	5	5	4	9	23 (34.8)

*Four cases lack of diagnostic imaging data, not included in the statistics

** Proportion of IMN in combination with local recurrence in relapsed population

Abbreviations:*IMN*, Internal mammary lymph node; *ALN* Axillary lymph node; *SCN*, supraclavicular node

#### IMNs in relation to IMVs

The maximal radial distance from center of metastatic nodes to the IMV in each patient were measured and documented. The percentage of including IMN with a 4 mm, 5 mm, 6 mm and 7 mm medial/lateral distance to the IMV were 54.2% (45/83), 75.9% (63/83), 89.2% (74/83) and 92.3% (77/83) respectively. While in maximal dorsal/ventral distance, all extension of the IMN nodes were in dorsal direction, the percentage of including IMN with a 4 mm, 5 mm, 6 mm and 7 mm depth to the IMV were 88%(73/83), 91.6% (76/83), 96.4%(80/83) and 98.8%(82/83) respectively, one case had enlarged mass invading the fourth rib, for which the measurement was difficult.

### Natural infiltration expansion of the IMNs progression

**Fig. 2:**
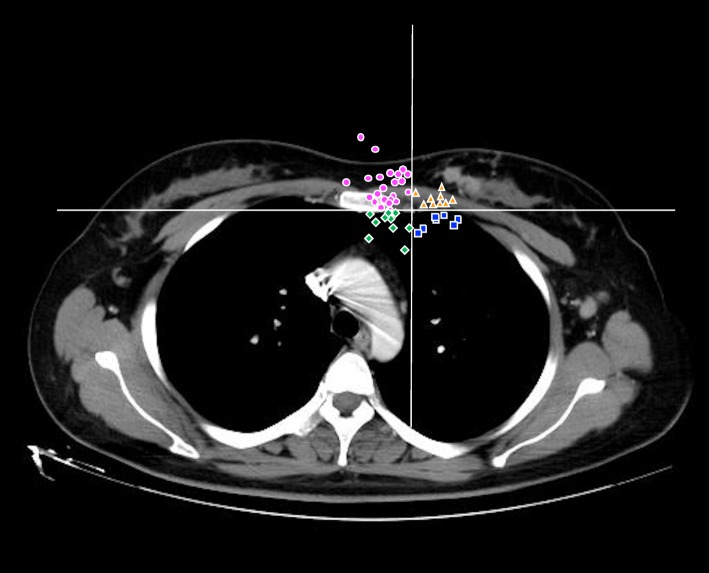
Natural expansion of the IMNs progression. More dots are located in the area near sternum

Overall, 49 cases with visible IMN adenopathy larger than 1 cm in diameter was evaluated to analyze the tendency of natural extension. All cases of IMNs were mapped manually using CT imaging of a female patient who had left-sided IMN relapse. Her CT scan of the chest with intravenous contrast acquired with both arms abducted overhead and uploaded onto the Eclipse planning system. After labeling the farthest point of IMN metastatic lesions on axial CT imaging, 32 patients (65.3%, 32/49) were found to have a growth direction towards sternum, and 23 of them (71.9%, 23/32) tend to spread above the sternum (Fig. 2). Invasion of the sternum was observed in 12 cases. Unfortunately, no significant difference was obtained with *P* value over 0.05(*P* = 0.38).

### Diagnostic rate of different imaging method

The diagnostic rates of enlarged IMN detected on US, CT, MR, and PET–CT are approximately 36.0% (18/50), 34.9% (22/63), 90.3% (28/31) and 13.0% (3/23), respectively. Most frequent description displayed as “parasternal mass/lump”and “chest-wall mass/lump” for CT diagnostic documents. The diagnostic efficiency of IMN can be increased to 67.4% if we combined different imaging methods.

## Discussion

Involvement of internal mammary nodes is considered to be an ominous prognostic factor,both in the setting of initial diagnosis and the recurrence. A large population-based study concluded that IMN recurrence could perform as a forerunner of metastatic disease after a long-term follow-up of 6000 breast cancer patients [11]. A most recent study mapping the anatomic pattern of isolated nodal recurrences demonstrated a relatively higher occurrence of IMN recurrence (32.5%) following axillary nodes (42%) with least recurrence of supraclavicular area (25.5%) compared with the rare recurrence rate from other studies [10]. It also presented as an portent for significantly inferior overall survival when compared with an axillary recurrence with no IMN involvement. For breast cancer patients with confirmed IMN metastases, studies have proven a definite improvement of clinical outcomes if irradiation to the internal mammary nodal chain was performed [31, 32]. Veronesi et al. has observed patients with positive but receiving IMNs radiotherapy would experience a considerable excellent survival compared with the patients with negative IMN [33]. However, it also raises the risk of radiation-induced complications especially for pneumonitis and ischemic heart disease and inevitably challenge the complexity of the treatment planning due to its immediately adjacent to these organs. This suggests that it is of great importance to delineate the target fields appropriately so that it can reduce the relapse as well as spare the organs at risk. Thus a better knowledge of IMN anatomical structure would be indispensable for radiation oncologist.

In this study, we not only detail the anatomical and imaging characteristics of the IMN by analyzing distribution regularities of involved ribs, distance from the IMV to the nodal center, but also calculate the incidence differences in the of IMN involvement between left and right laterality. In particular, we try to figure out the natural extension of IMNs from IMV which would provide more information for the CTV delineation of visible IMN mass. Our results ultimately show that the location of the IMN metastasis was mostly observed in the first, second, and third intercostal spaces with few occurring caudal to the fourth rib and none in the fifth. Among those, the second intercostal space was the most likely involved in patients with single or multiple intercostal space involvement. This was consistent with other reports from extended radical mastectomy and autopsy studies, as well as from radiological images reviews that the vast majority of IMN recurrence were located within the first three intercostal spaces [2, 6, 34]. Jethwa et al. also reported only 14% of lymph nodes were located in the fourth and fifth intercostal spaces among 130 invisible IMN metastases [35]. One study mentioned 100% IMN metastases occurred to the first to third intercostal spaces in the series of 61 patients who underwent extended radical mastectomy [10]. Nowadays, most of the current delineation guidelines for prophylactic IMN CTV limits the caudal border at the cranial edge of the fourth rib [21–24]. Our results, together with the aforementioned data have reaffirmed the rationality of the recommendations for prophylactic IMN delineation. But what is noteworthy is that in our study, four IMNs located at the forth int [35] ercostal space were extending from the upper one, indicating a rare prevalence of isolated IMN involvement in the fourth let alone fifth intercostal spaces in the absence of cranial IMN involvement in the IMN chain. That is to say, it would be reasonable to extend the caudal border in the cases with clinical IMN involvement in the first three intercostal spaces for prophylactic delineation.

Currently, most published guidelines, such as PROCAB, DBCG, and ESTRO guidelines, recommend a 5-mm medial and lateral expansion on the IM vessels to delineate the clinical target volume of internal mammary nodal chain, while RTOG guideline suggest the IMN target volume encompasses the IM vessels alone. Another study suggested that medial and lateral expansions as large as 6 mm would have encompassed 95% of IMNs for patients with known IMN involvement and those at very high risk of harboring microscopic disease via the analysis of 67 patients with IMN disease [35]. In our study, only 75.9% of lymph node could be encompassed with a 5-mm medial/lateral expansion on the IMV. Therefore, a 7 mm medial/lateral expansion to IMV might be appropriate to delineate the CTV of IMN, which covers 90% of IMNs, and a 6 mm expansion in the dorsal or ventral direction would be safe to include about 95% cases. The recommended medial and lateral expansion in the present study is larger than the current guidelines, one possible reason for this finding is the potential selection bias, because nearly 90% of the patients had larger IMN adenopathy in our series. Nevertheless, our finding is in accordance with the guideline enforced in our center with satisfied dose controlling to the heart and lungs.

Unlike anatomical features mentioned above, very few studies have focused on the natural extension of the internal mammary lymph nodes. It is generally acknowledged that tumors would always tend to grow in the direction with less resistance to acquire a fully extension. Interestingly, we found that 65.3% of the lesions expanded inwardly, that is, towards sternum side, which is different from the assumption that creeping along pleura towards the pulmonary lobe would provide more space for tumor growth. At present, CTV is obtained from gross tumor volume for visible IMN metastases especially among patients with subsequent IMN relapse. It is conceivable that areas at risk of harboring microscopic disease may not be fully congruent with areas harboring macroscopic disease. Given the situation that there is no specific consensus regarding the microscopic disease of metastatic IMN, the optimal CTV boundary has not been developed. From this point of view, our data has an obvious clinical significance suggesting that a deliberately appropriate expansion of CTV towards the sternum side would achieve a better disease control for those with clinical IMN adenopathy. Consistent with our study, a recent report also mentioned IMN abutting the sternum and suggested to extend CTV coverage all the way to the sternal border [34, 36]. Interestingly, we observed a majority of IMNs (47/76, 61.8%) was located on the left and had more chance to get larger volume. A recent report from Japan has obtained the similar result [36]. Another latent proof is DBCG-IMN trial from which an obvious overall survival benefit but equal cardiac events have been acquired for patients with IMN irradiation on the right side compared with those without IMN irradiation on the left. However, further study with larger sample size and fundamental or translational researches are still needed to validate and confirm our findings.

Additionally, imaging plays a pivotal role in the management of cancer patients, including diagnosis, initial staging and treatment response assessment. MRI is the most efficient method, it is often used for initial diagnosis. While CT may be the most widely used tool throughout the treatment process, especially in patients with recurrence and metastasis, thus it is critical to improve the diagnostic accuracy. However, CT is prone to have missed diagnosis in cases with sternal metastases or chest wall recurrence. At the same time, as a non-invasive method, ultrasound plays an important role in detecting positive nodes to improve the diagnosis rate. When all inspection methods are used in combination, the diagnostic efficiency of IMN can be increased to 67.4%. Therefore, multimodal imaging was recommended to early detect abnormal IMN.

Several limitations are needed to be concerned in our study. First of all, positive IMNs were determined based on radiologic criteria and only 16.9% (14 of 83) of our patients had biopsy confirmation of their IMN metastasis. Secondly, our study is a retrospective study, thus the inherent selection bias could not be excluded. Finally, the sample size of the study is relatively small.

## Conclusions

In conclusion, we mapped the location of IMN metastases to assist in optimizing the IMN CTV for volume-based breast cancer treating planning. In most of the patients indicated for IMN irradiation in the adjuvant setting, clinical target volume of IMN could be delineated with a 7 mm medial and 6 mm dorsal distance to the IMV on the same axial CT image which is in accordance with the recommendation in our center. In patients with evident metastatic IMNs, deliberate delineation towards sternum might be reasonable in terms with the natural extension orientation of IMN. This optimization in delineation might facilitate the balance of target coverage and normal tissue constraints. Multi-imaging mode recommended to improve the diagnostic specifity and sensitivity of IMN metastases.

## Data Availability

The datasets supporting the conclusions of this article are included within the article.

## Abbreviations

ALN: Axillary lymph nodes
CTV: Clinical tumor volume
FNA: Fine-needle aspiration
IMN: Internal mammary lymph nodes
IMRT: Intensity-modulate radiotherapy
IMV: Internal mammary vessel
RNI: Regional node irradiation
